# Phenotypic and genotypic characterization of clinical *Staphylococcus aureus* isolates from Kenya

**DOI:** 10.1186/s12866-019-1597-1

**Published:** 2019-11-06

**Authors:** Cecilia Kyany’a, Justin Nyasinga, Daniel Matano, Valerie Oundo, Simon Wacira, Willie Sang, Lillian Musila

**Affiliations:** 1 Kenya Medical Research Institute, P. O. Box 54840-00200, Nairobi, Kenya; 2 The United States Army Medical Research Directorate-Africa, P.O. Box 606-00621, Village Market, Nairobi, Kenya; 3 Technical University of Kenya, P.O. Box 52428-00200, Nairobi, Kenya

**Keywords:** MRSA, MSSA, Kenya, Virulence, Resistance, Genome

## Abstract

**Background:**

The increase and spread of virulent-outbreak associated, methicillin and vancomycin resistant (MRSA/VRSA) *Staphylococcus aureus* require a better understanding of the resistance and virulence patterns of circulating and emerging strains globally. This study sought to establish the resistance phenotype, and strains of 32 non-duplicate clinical MRSA and MSSA *S. aureus* isolates from four Kenyan hospitals*,* identify their resistance and virulence genes and determine the genetic relationships of MRSA with global strains.

**Methods:**

Antimicrobial susceptibility profiles were determined on a Vitek 2, genomic DNA sequenced on an Illumina Miseq and isolates typed in-silico. Resistance and virulence genes were identified using ARIBA and phylogenies generated using RAxML.

**Results:**

The MRSA isolates were 100% susceptible to vancomycin, teicoplanin, linezolid, and tigecycline. Nine distinct CC, 12 ST and 15 spa types including the novel t17826 and STs (4705, 4707) were identified with CC8 and CC152 predominating. MRSA isolates distributed across 3 CCs; CC5-ST39 (1), CC8 – ST241 (4), a novel CC8-ST4705 (1), ST8 (1) and CC152 (1). There was > 90% phenotype-genotype concordance with key resistance genes identified only among MRSA isolates: *gyrA*, *rpoB*, and *parC* mutations, *mecA*, *ant (4′)-lb, aph (3′)-IIIa, ermA, sat-4, fusA, mphC* and *msrA*. Kenyan MRSA isolates were genetically diverse and most closely related to Tanzanian and UK isolates. There was a significant correlation between *map*, *hlgA*, *selk*, *selq* and *cap8d* virulence genes and severe infections.

**Conclusion:**

The findings showed a heterogeneous *S. aureus* population with novel strain types. Though limited by the low number of isolates, this study begins to fill gaps and expand our knowledge of *S. aureus* epidemiology while uncovering interesting patterns of distribution of strain types which should be further explored. Although last-line treatments are still effective*,* the potential for outbreaks of both virulent and resistant strains remain, requiring sustained surveillance of *S. aureus* populations.

## Background

*Staphylococcus aureus* is a gram-positive bacterium responsible for a broad spectrum of clinical infections ranging from benign skin rashes to necrotizing tissue and pulmonary lesions. The increasing prevalence of multidrug-resistant methicillin and vancomycin-resistant *S. aureus* strains (MRSA and VRSA) limit available therapeutic options making these infections challenging to manage. Since the emergence of methicillin resistance (MRSA) in the 1940s, epidemics caused by successful MRSA [1, 2] have been observed, e.g. USA 300, a highly virulent MRSA strain that emerged in the USA and is currently associated with community outbreaks globally [3] and E-MRSA 15 which emerged in the UK and caused various hospital outbreaks [4]. The clonal success is attributed to factors that enhance binding to host tissues and the acquisition of virulence genes, e.g., USA 300 which has acquired the arginine catabolic mobile element, *sek* and *seq* virulence genes [5–7]. Nosocomial outbreaks of MRSA are frequent in daycare centers, nursing homes, and critical care units [8] and with the emergence of more virulent CA-MRSA [9], *S. aureus* outbreaks can significantly increase morbidity and mortality.

*S. aureus* can exhibit resistance to several antibiotics due to genes encoded on the chromosome and the acquisition of resistance genes by horizontal transfer of individual genes or resistance islands from other *S. aureus* isolates and other bacterial species, e.g., *Van* genes acquired from Vancomycin-resistant enterococci (VRE) on mobile elements [10]. The implications of acquired drug resistance in bacteria to public health are profound. In Kenya, previously manageable diseases such as typhoid and cholera have caused health crises due to the emergence of highly drug-resistant strains of H58 *Salmonella typhi* [11] and *Vibrio cholera* [12, 13] that quickly dominate endemic antibiotic sensitive strains both locally and globally [14]. Monitoring of emerging resistance patterns and genes is therefore essential for the management of new resistant strains with outbreak potential.

Phenotypic testing is the gold standard for determining the antibiotic susceptibility of a bacterium [15] but is limited by the number of antibiotics one can reasonably test in the laboratory. Genomic analysis is ideal for detecting resistance against multiple antibiotics, identifying new resistance genes, mutations or new synergistic relationships affecting resistance genes. Genomic testing is best when used in tandem with existing phenotypic data since it can be challenging to predict resistance based solely only on the presence or absence of resistance genes [16, 17] due to redundant antibiotic resistance mechanisms and the impact of mutations in resistance genes, modifiers of gene expression and accessory genes on phenotypic resistance.

Multilocus sequence typing (MLST) and typing of the staphylococcal protein A (*spa*) gene have been widely used to identify different *S. aureus* strain types [18, 19]. For MRSA, typing of the staphylococcal cassette chromosome (SCC*mec*) which harbors the gene encoding methicillin resistance provides additional strain discrimination [20–22]. Studies on *S. aureus* isolates from Kenya have focused mainly on antimicrobial susceptibility testing [23, 24] and strain typing with limited testing for resistance genes and virulence determinants which can influence infection severity. The virulence genes reported in the few Kenyan studies looking at both *S. aureus* infections and carriage include Panton-Valentine leukocidin (*pvl*), Toxic shock syndrome (*tsst-1*), exfoliative toxin A and enterotoxin A with a notably high prevalence of *pvl* reported [25, 26]. These studies were, however, limited to four healthcare institutions in close geographic proximity. Therefore, there is limited information on the genomic diversity and distribution of the *S. aureus* population across Kenya.

This study sought to fill this gap by characterizing both resistance and virulence determinants of clinical isolates from different geographical areas in Kenya and identifying the relationships between Kenyan MRSA strains and known global strains. By broadening our understanding of the *S. aureus* population in Kenya, this study provides baseline epidemiological data on the type distribution, drug resistance patterns and emerging virulent strains in Kenya.

## Results

Of the 17 antibiotics tested only 16 had complete results for all isolates. All isolates were resistant to at least one of the drugs in the panel of 16 antibiotics analyzed. No resistance was detected against vancomycin, teicoplanin, tigecycline or nitrofurantoin in any isolate while all isolates were resistant to penicillin (Additional file 1). Eight isolates were classified as MRSA and confirmed to possess the *mecA* gene by PCR.

There was high sensitivity to vancomycin, linezolid, teicoplanin, nitrofurantoin and tigecycline among all isolates. The MRSA isolates were multidrug resistant with 100% resistance to erythromycin, oxacillin, cefoxitin and had varied susceptibilities to rifampicin (50%) and < 25% susceptibility to the remaining drugs tested. In contrast, among the 24 MSSA isolates, > 70% were susceptible to a majority of antibiotics tested with reduced susceptibility (< 75%) to trimethoprim (71%) and rifampicin (67%) (Additional file 1).

**Fig. 1:**
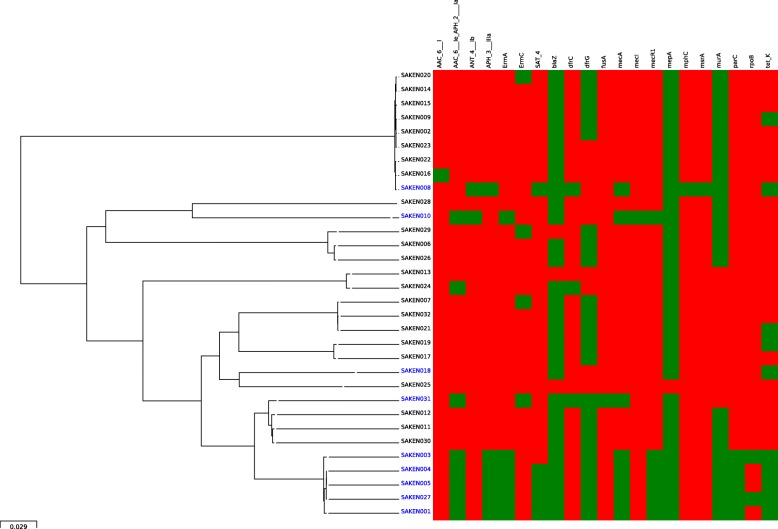
Core genome SNP phylogeny and heat map of antibiotic resistance genes identified. MRSA isolates are shown in blue and MSSA isolates are in black. Green denotes the presence and red the absence of the genes listed. Ubiquitously expressed genes are not indicated (*S. aureus 23S, arlR, arlS, mgrA,* and *tet38*)

Resistance genes identified are listed in Additional file 1, and their distribution is shown on the heat map in Fig. 1. Phenotype-genotype concordance of 90–99% was observed. Discordance was observed for rifampicin and aminoglycosides drug classes. The genes *23S rRNA* and *tet38,* important in macrolides and tetracycline resistance [27, 28] and the multigene regulators important for multidrug resistance and virulence gene expression *arlR/arlS, mgrA* [29–31] were ubiquitously expressed among the isolates and are not indicated on the figure. All the MRSA isolates had *mecA* mediated methicillin resistance. The multidrug resistant phenotype of the MRSA isolates was supported by the presence of multiple antibiotic resistance genes which varied in number and composition by the MRSA lineages (Fig. 1). The resistance genes detected among MRSA ranged from 9 to 14 compared to 1–5 for MSSA isolates. The genes associated only with MRSA isolates were the *ant (4′)-lb, aph (3′)-IIIa, ermA, sat-4*, *fusA*, *mphC*, *msrA* genes, the quinolone resistance-conferring mutations on *parC* (S80F) and *gyrA* (S84 L) and the rifampicin resistance mutations in *rpoB*. *TetK*, *dfrG,* and *dfrC* were found among both MSSA and MRSA.

Isolate typing using the various methods indicated great diversity among the isolates with identification of 9 distinct clonal complexes (CC5, 8, 15, 22, 80, 88, 121, 152, 580), 12 ST types (ST8, 15, 22, 39, 80, 121, 152, 241, 580, 1633, 4705, 4707), 15 *spa* types (t005, t007, t037, t064, t084, t186, t272, t314, t355, t1476, t2029, t4198, t5941, t13194 and a novel *spa* type, assigned t17826) (Table 1). Among the MRSA, three known staphylococcal cassette chromosome types, SCC*mec*_type2A, 3A and 2B and a novel divergent SCC*mec* element were identified.

**Table 1:** Table of isolate characteristics

	Isolate ID	Clonal complex	ST	*spa* type	SSC *mec*	REGION	CAI/HAI^b^	IP/OP
**MRSA**	**SAKEN001**	**8**	**241**	**37**	**SCC** ***mec*** **_type_III (3A)**	**Kisumu**	**CAI**	**In-patient**
	**SAKEN003**	**8**	**4705** ^**a**^	**2029**	**SCC** ***mec*** **_type_III (3A)**	**Kisumu**	**CAI**	**Outpatient**
	**SAKEN004**	**8**	**241**	**37**	**SCC** ***mec*** **_type_III (3A)**	**Kisumu**	**HAI**	**In-patient**
	**SAKEN005**	**8**	**241**	**37**	**SCC** ***mec*** **_type_III (3A)**	**Kisumu**	**CAI**	**In-patient**
	**SAKEN008**	**152**	**152**	**355**	**SCC** ***mec*** **_type_IVa (2B)**	**Nairobi**	**CAI**	**Out-patient**
	**SAKEN010**	**5**	**39**	**7**	**SCC** ***mec*** **_type_II (2A)**	**Kisumu**	**CAI**	**Out-patient**
	**SAKEN027**	**8**	**241**	**37**	**SCC** ***mec*** **_type_III (3A)**	**Kisumu**	**HAI**	**In-patient**
	**SAKEN031**	**8**	**8**	**1476**	**novel cassette:** ***mecA*** **present**	**Nairobi**	**CAI**	**Out-patient**
MSSA	SAKEN017	15	15	84	n/a	Kericho	CAI	Out-patient
	SAKEN024	22	22	5	n/a	Kericho	CAI	Out-patient
	SAKEN032	80	80	13,194	n/a	Kericho	CAI	Out-patient
	SAKEN026	121	121	314	n/a	Kericho	CAI	Out-patient
	SAKEN009	152	152	355	n/a	Kericho	CAI	In-patient
	SAKEN016	152	152	355	n/a	Kericho	CAI	Out-patient
	SAKEN025	5	4707^a^	17826^a^	n/a	Kisumu	CAI	Out-patient
	SAKEN030	8	8	unknown	n/a	Kisumu	CAI	In-patient
	SAKEN019	15	15	unknown	n/a	Kisumu	CAI	In-patient
	SAKEN018	88	88	186	n/a	Kisumu	CAI	Out-patient
	SAKEN029	121	121	272	n/a	Kisumu	CAI	Out-patient
	SAKEN002	152	152	355	n/a	Kisumu	CAI	Out-patient
	SAKEN011	8	8	64	n/a	Kisumu	CAI	In-patient
	SAKEN012	8	8	64	n/a	Kisumu	CAI	In-patient
	SAKEN021	80	80	13,194	n/a	Kisumu	CAI	Out-patient
	SAKEN020	152	152	355	n/a	Kisumu	CAI	In-patient
	SAKEN028	580	580	unknown	n/a	Malindi	CAI	Out-patient
	SAKEN013	22	22	84	n/a	Nairobi	CAI	Out-patient
	SAKEN007	80	80	5941	n/a	Nairobi	CAI	In-patient
	SAKEN006	121	121	4198	n/a	Nairobi	CAI	In-patient
	SAKEN014	152	152	355	n/a	Nairobi	HAI	In-patient
	SAKEN015	152	1633	355	n/a	Nairobi	HAI	In-patient
	SAKEN022	152	152	355	n/a	Nairobi	CAI	Out-patient
	SAKEN023	152	152	355	n/a	Nairobi	CAI	In-patient

^a^denotes a novel ST and *spa* type. ^b^*CAI* community-acquired infection, *HAI* hospital-acquired infection. MRSA isolates are shown in bold

A majority of the isolates belonged to CC152 (9/32) and CC8 (9/32). MRSA in this study classified as CC5, 8 and 152 with a majority (4/9) belonging to ST241 and *spa* type t037. Two novel STs assigned ST4705 (CC8, MRSA) and ST4707 (CC5, MSSA) by PubMLST [32] were reported. *Spa* typing indicated t355 as the dominant *spa* type (9/32; 28.1%). The eight MRSA isolates belonged to 5 *spa* types; t007 (1), t037 (4), t2029 (1), t1476 (1) and t2029 (1). MSSA isolates had greater *spa* diversity than MRSAs with t005, t186, t314, t4198, t5941 represented by single isolates.

Some strain types were found in only particular geographical regions, for example, ST88 and ST241 detected in Kisumu County only, in contrast to CC152 which showed a wide geographic distribution. Isolates from Kericho County were the most heterogeneous based on CC/ST types.

**Fig. 2:**
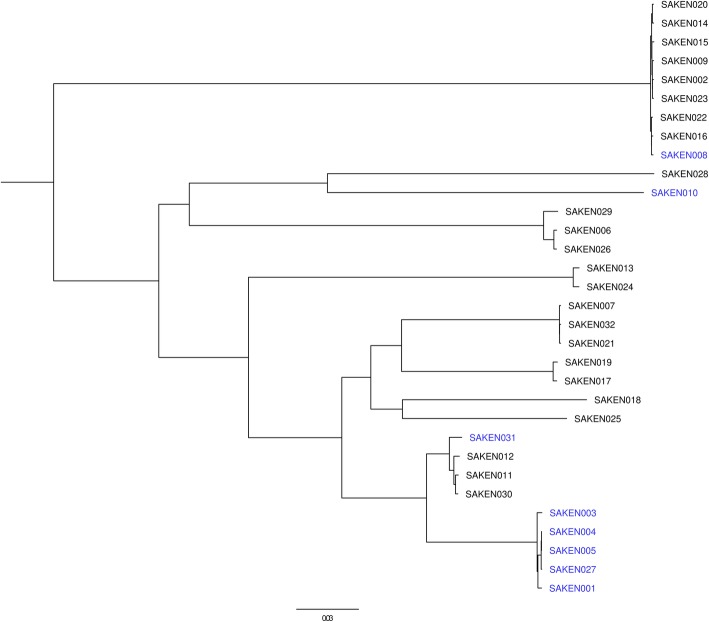
Core genome SNP phylogeny of Kenyan *S. aureus* isolates. MRSA isolates are in blue, and MSSA isolates are in black. MRSA isolates are distributed across four clusters

**Fig. 3:**
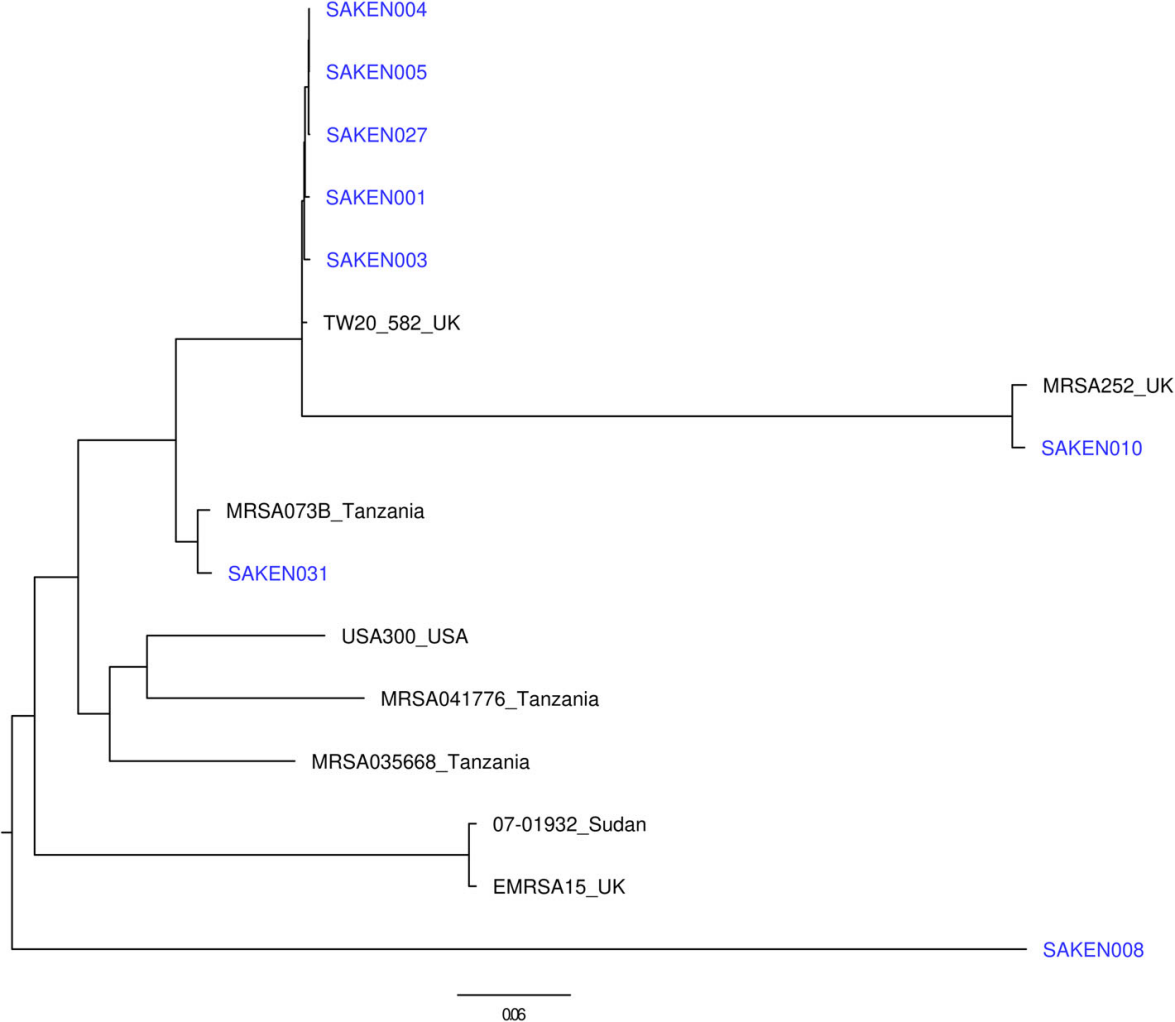
Core genome phylogeny of MRSA isolates from this study (in blue) and known global and regional strains. Although the isolates are genetically diverse, Kenyan MRSA isolates are closely related to Tanzanian ad UK strains

Phylogenetic analysis of the Kenyan isolates indicated SNP differences of 5–27,562 SNPs and clustered the isolates by ST or CC types. MRSA isolates were distributed across four clusters. The largest MRSA cluster consisting of SAKEN1, 3, 4, 5, and 27 clustered within CC8 with MSSA isolates. SAKEN004 and SAKEN005 isolates had only 5 SNP differences and were from patients admitted in the same hospital at the same time. SAKEN004, 005,027 had SNP differences between 5 and 34 SNPs, an indication that they were closely related but distinct isolates (Fig. 2). Kenyan MRSA isolates were genetically diverse and most closely related to MRSA073B from Tanzania, MRSA252 UK, and TW20_582_UK (Fig. 3).

**Fig. 4:**
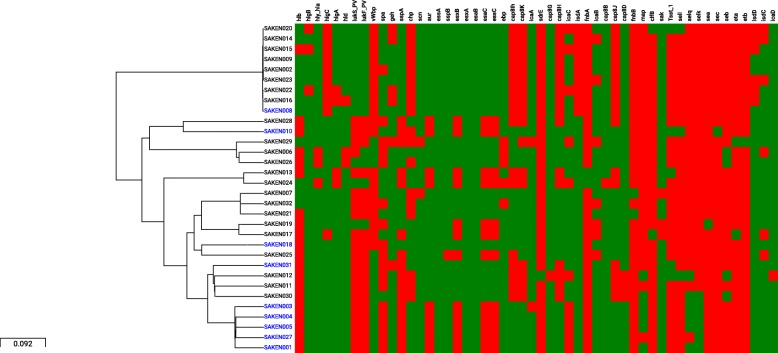
Core genome phylogeny and a heat map showing the distribution of virulence genes among study isolates. Green denotes the presence and red indicates the absence of the gene. Virulence factors are grouped according to function. Genes that were ubiquitously expressed among the isolates are not shown. MRSA isolates are shown in blue and MSSA isolates are in black

The virulence genes identified among the isolates are indicated in Fig. 4 and grouped according to function; pore formers, immune evasion, toxins, and adhesins. There was no significant difference in the numbers of virulence genes between MRSA and MSSA isolates (*p* = 0.09), but there was a significant association (*p* < 0.05) between the severity of the infection and the five virulence genes; *map*, *hlgA*, *selk*, *selq,* and *cap8d*. *Map, selk, selq, hlgC, vwbp* virulence genes were significantly associated with CC8 (*p* < 0.00005) but showed varied distribution within the CC. There was a strong association between isolates in CC152 and the presence of *LukS_PV, LukF_PV and hlb* and the absence of *hlgc*, *vwbp*, *capIh*, *chp*, i*sdA*, *isdD*, *cap8h* and *cap8K* (*p* < 0.00005).

## Discussion

In this study, antimicrobial resistance phenotypes and genotypes and strain types of *S. aureus* isolates from diverse geographical areas in Kenya were investigated and phylogenetic relationships inferred between the isolates and known global and regional strains based on whole genome sequences. Virulence genes were identified, and their presence relative to the clonal complexes and clinical presentation examined for possible correlations to identify genetic predictors of hyper-virulence.

Phenotypic antimicrobial susceptibility testing indicated high sensitivity to vancomycin, linezolid, teicoplanin, nitrofurantoin and tigecycline among all isolates consistent with previous observations by Gitau et al. [33]. High levels of resistance to benzylpenicillin, sulfamethoxazole, rifampicin, tetracycline, and erythromycin have been reported by previous Kenyan [23, 25] and African [34] studies. The high levels of resistance to these commonly used antibiotics could be linked to Kenya having a high prevalence of tuberculosis (TB), HIV and malaria. Rifampicin is among the first-line agents for the treatment of TB [35] infections in Kenya which is linked to HIV infection [36] and is also used as an antimalarial drug. The MRSA isolates bearing the mutation in the *rpoB* gene conferring resistance to rifampicin were from Kisumu County which is endemic for malaria and has a high prevalence of HIV [37]. Previous Kenyan studies have reported SXT resistance rates of 40% [25] and 62% [23]. In this study, the high-level resistance (75%) to sulfamethoxazole (SXT), mediated by both chromosomally encoded dfrC and plasmid-borne dfrG genes may be driven by the use of SXT in HIV prophylaxis [37–39].

MSSA isolates were 100% susceptible to quinolones consistent with the high susceptibility rates indicated in other Kenyan studies [40–42] while MRSA isolates were 25% susceptible, markedly lower than a previous study that reported a susceptibility of 55.9% to ciprofloxacin among Kenyan MRSA [43] suggesting growing resistance of MRSA to quinolones in Kenya. Quinolone resistance was mediated by the resistance-conferring mutations on *parC* (S80F) and *gyrA* (S84 L) [44].

This study reports 62.5% susceptibility to tetracyclines which is higher than the 20–50.3% reported in Kenya, and other African studies [42, 45]. The previously observed increase in resistance to tetracycline in Africa (< 75%) was linked to increased use of tetracycline in animal husbandry [46], but the reduction in resistance observed in this study among human clinical isolates could be an indicator that tetracycline is being used less to treat human infections. *TetK* and *TetM* genes are reported [42] to be predominant in Sub-Saharan Africa, but this study has shown that TetK is the principal mediator of resistance and could be useful as a marker to monitor tetracycline resistance in Kenya.

Strain typing revealed 12 STs and 9 CCs among the isolates confirming the considerable heterogeneity previously described among *S. aureus* both regionally and globally [25, 34, 47, 48]. *Spa* typing showed higher discriminatory power than ST with multiple *spa* types belonging to the same STs. Of the major lineages described for MRSA, MRSA isolates in the present study belonged to CC8 and CC5, both of which are associated with global outbreaks [1]. CC8 was composed of both MSSA and MRSA isolates. CC5/ST241, t037 and ST 239, t037 are the predominant MRSA clones described in previous studies on Kenyan isolates from Nairobi and its environs [25, 47]. In this study, a majority of MRSA isolates also belonged to ST241, t037 even though they were from sites in Western Kenya situated ~ 300 km from Nairobi suggesting a widespread geographical distribution of the CC5/ST241 MRSA strain in Kenya. Schaumburg et al. [34] reported ST 241 MRSA clone to also be prevalent in Africa though with varying SSC*mec* types; Senegal (SSC*mec* III), Tunisia (SSC*mec* III), Niger (SSC*mec* III and V) Nigeria (SSC*mec* III and IV) and Algeria (SSC*mec* III) [49]. Even though globally most HAI-MRSA are SCC*mec* type I-III and CAI-MRSA SCC*mec* types IV and V [50, 51], the hospital associated strain ST 241 SCC*mec* III [49] was identified in both HAI and CAI infections in the present study and a previous Kenyan study [47]. SCC*mec* typing may, therefore, have limited utility as a marker of CAI or HAI in the region.

The Kenyan isolates grouped distinctly into several clonal complexes. The CC8 cluster was composed of two clades ST8 (MSSA) and ST241 (MRSA) with the two clades sharing a recent common ancestor. Studies have shown that MSSA isolates of CC8 act as reservoirs for MRSA pending acquisition of the staphylococcal cassette [52, 53]. Relationships between isolates of this study and known global strains using core genome SNPs revealed close clustering of a majority of MRSA strains in this study with the well-known strains. The predominant CC8 MRSA isolates in this study were closely related to the CC8 TW20 strain 582, which is a successful HAI MRSA clone from London [4], known for its high transmissibility and multi-drug resistant properties due to a plethora of resistance genes carried on mobile elements [54]. CC8 MRSA strains have been linked to community-acquired infections and include other well-known strains such as USA 300 which is a lineage-linked to the acquisition of SSC*mec* IV, *pvl* and *seq* and *sek* genes [5, 55]. MRSA isolates in this study were also closely related to MRSA252 from UK and MRSA_0411776 from Tanzania indicating the ease of spread of *S. aureus* strains across regional and international borders.

There was a significant correlation between the five virulence genes: *map*, *hlgA*, *selk*, *selq* and *cap8d* and severe infections indicating their potential usefulness as markers of infection severity in the region. While CC8 isolates were strongly associated with the presence of multiple virulence genes, CC152 was in contrast associated with an absence of these virulence genes but the presence of *pvl*, a bi-component leukocidin (lukF_PV and lukS_PV) destroying leukocytes and causing tissue necrosis. *Pvl* predominant ST152 clones have been described in Nigeria [56] and Mali [57] and Europe [58] indicating a global distribution of this clone. The MRSA prevalence in Kenya ranges widely from 3 to 30% [24, 33], and although, as this study has shown, most *S. aureus* infections remain relatively easy to treat, the morbidity associated with hypervirulent strains could be managed better by understanding the circulating strains and their virulence gene profiles.

Despite the low sample numbers, this study does begin to fill gaps and expand our understanding of the epidemiology of *S. aureus* by providing data on clinical isolates of *S. aureus* from other parts of the country as previous studies in Kenya have been limited to four healthcare institutions within close geographic proximity. The isolates in this study, collected over a 3-year period (2015 to Aug 2018), uncover patterns of distribution of different strain types that are interesting and will be explored further as part of the ongoing surveillance to examine whether the observed *S. aureus* distribution patterns hold and other patterns emerge over time with more isolates.

## Conclusion

This study provides insight into the diversity, distribution and resistance profiles of Kenyan MSSA and MRSA isolates and their relatedness to global MRSA strains. Although limited by the low numbers of isolates this study provides a baseline for monitoring *S. aureus* strain types and associated resistance and virulence patterns to create risk maps for *S. aureus* infections in Kenya. The study has identified multidrug resistance genes carried by Kenyan *S. aureus* isolates and provided a basis to track trends in drug resistance and identify emerging resistance patterns and novel strain types. The evidence of co-occurrence of methicillin resistance and virulence genes portend the emergence of highly virulent MRSA infections that could be outbreak-associated. In the advent of increasing drug resistance in Kenya, continued surveillance using both phenotype and genotype data is recommended to identify country-specific data on drugs effective for treatment for both MRSA and MSSA to reduce morbidity given the unique backdrop of other endemic diseases.

## Materials and methods

### Bacterial isolates identification and drug susceptibility testing

Non- duplicate clinical *S. aureus* isolates from patients enrolled in an ongoing surveillance study (WRAIR#2089, KEMRI#2767) in four hospitals in Kisumu, Kericho, Malindi, and Nairobi counties in Kenya were analyzed for this study. *S. aureus* isolates were identified based on characteristic beta hemolysis, catalase, and coagulase positive phenotypes. Isolate identity was confirmed and antimicrobial susceptibility testing performed on the Vitek 2 platform (bioMérieux, Hazelwood, MO, USA) using the GP identification and the P580 antibiotic susceptibility card which tests a panel of 17 drugs (Benzylpenicillin, oxacillin, gentamicin, tobramycin, levofloxacin, moxifloxacin, erythromycin, clindamycin, linezolid, teicoplanin, vancomycin, tetracycline, tigecycline, nitrofurantoin, fusidic acid, rifampicin, trimethoprim/sulfamethoxazole). MRSA was identified using CLSI break-points for oxacillin MIC and cefoxitin screen and validated by PCR for the presence of the *mecA* gene using published primers [22]. All MRSA isolates identified between April 2015 and August 2018 and a selection of methicillin susceptible *S. aureus* (MSSA) isolates from each county totaling 32 isolates (8 MRSA, 24 MSSA) were included in this study. The isolates were from both in- and out-patients. The infections were classified as severe if the patient was admitted in the hospital (17/32, 53.2%) or as mild if they were treated in the out-patient department (15/32, 46.9%). 87.5% (28/32) of the isolates were from patients with community-acquired infections and 12.5% (4/32) with hospital-acquired infections as per the CDC classification [59] (Table 1).

### Whole genome sequencing and sequence analysis

Genomic DNA was extracted from freshly cultured *S. aureus* isolates using the ZR Fungal/Bacterial DNA MiniPrep Kit (Zymo research, California, United States). DNA concentrations were determined using the Qubit (Thermo Fisher Scientific, Massachusetts, United States) and 1 ng of DNA used for library preparation with the Nextera XT kit (Illumina Inc. San Diego, California, United States) as per manufacturer’s instructions to generate 300 bp paired-end libraries. Libraries were sequenced on an Illumina MiSeq platform (Illumina Inc. San Diego, California, United States). Raw reads were trimmed for quality and de-novo assembly performed using Newbler [60]. Genome assemblies were uploaded onto NCBI under BioProject ID PRJNA481322.

### Isolate typing

In-silico *spa* typing was performed on assembled genomes using SpaTyper 1.0 hosted on the Centre for Genome Epidemiology (CGE) https://cge.cbs.dtu.dk/services/spatyper/ [61]. In-silico MLST sequence type (ST) were obtained on https://cge.cbs.dtu.dk/services/MLST/ [62] at the Centre for Genomic Epidemiology and isolates grouped into clonal complexes using the BURST clustering algorithm available on http://eburst.mlst.net/, allowing a minimum of 6 identical loci for group definition. Sequences of novel STs were submitted to https://pubmlst.org/saureus/ for ST assignment [63]. Staphylococcal cassette types for the MRSA isolates were determined in-silico using SCCmecFinder 1.2 hosted on https://cge.cbs.dtu.dk/services/SCCmecFinder/ [64].

### Antimicrobial resistance and virulence genes identification

Genes coding for antimicrobial resistance and virulence were identified using ARIBA [65] (version 2.11.1) employing CARD [66] (version 3.0.1) https://card.mcmaster.ca as the reference database. To investigate the presence of virulence factors, the whole genomes of the Kenyan isolates were screened for 85 known virulence genes using the Virulence Factors Database (VFDB). AMR gene distribution and heat maps were generated and visualized on Microreact at https://microreact.org/ [67].

Phylogenetic analysis was performed to infer relationships between the eight Kenyan MRSA and eight known global strains; selected to include at least one whole genome for all the sequence types identified in the Kenyan isolates. The reference strains used in the phylogenetic analysis are listed in the Additional file 2. High-quality SNPs were called, and maximum likelihood phylogeny inferred using RAxML [68].

## Supplementary information

**Additional file 1.** Table of susceptibility testing results obtained from the Vitek 2 platform and genotypes detected using ARIBA. MICs were interpreted using the CLSI guidelines and expert deductions on the Vitek Advanced Expert System (AES). * indicates forced resistance by the AES. **denotes presence of only the ubiquitous resistance genes in the isolates (*S. aureus* 23S, *arlR*, arlS, *mgrA* and *tet38*). Isolates in bold are confirmed MRSA validated as *MecA* positive by PCR testing. Fusidic acid is not shown as results were missing for some isolates.

**Additional file 2.** List of reference genomes used in this study.

## Data Availability

The datasets used and/or analysed during the current study are available from the corresponding author on reasonable request. Sequence data is available on NCBI under BioProject ID PRJNA481322.

## Abbreviations

AES: Advanced Expert System
AFHSB: Armed Forces Health Surveillance Branch
ARIBA: Antimicrobial Resistance Identification By Assembly
CA: Community acquired
CAI: Community acquired infection
CARD: Comprehensive Antibiotic Resistance Database
CC: Clonal complex
CGE: Center for Genome Epidemiology
CLSI: Clinical and Laboratory Standards Institute
DNA: Deoxyribonucleic acid
GEIS: Global Emerging Infections Surveillance and Response System
HAI: Hospital acquired infection
IRB: Institutional review board
KEMRI: Kenya Medical Research Institute
MIC: Minimum inhibitory concentration
MLST: Multi-locus sequence typing
MRSA: Methicillin resistant *Staphylococcus aureus*
MRSN: Multidrug Resistance Surveillance Network
MSSA: Methicillin susceptible *Staphylococcus aureus*
PCR: Polymerase chain reaction
PROMIS: Proposal Management Information System
*Pvl*: Panton-Valentine leukocidin
RAxML: Randomized Axelerated Maximum Likelihood
SCC*mec*: Staphylococcal cassette chromosome
SNPs: Single nucleotide polymorphisms
Spa: *Staphylococcus aureus* Protein A
ST: Sequence type
SXT: Sulfamethoxazole
TB: Tuberculosis
*tsst-1*: Toxic shock syndrome
UK: United Kingdom
Van: Vancomycin
VFDB: Virulence Factors Database
VRE: Vancomycin resistant enterococci
VRSA: Vancomycin resistant *Staphylococcus aureus*
WRAIR: Walter Reed Army Institute of Research
